# Development and validation of a new assessment tool for suturing skills in medical students

**DOI:** 10.1007/s00238-017-1378-8

**Published:** 2017-12-04

**Authors:** Henriette Pisani Sundhagen, Stian Kreken Almeland, Emma Hansson

**Affiliations:** 1 Department of Plastic and Reconstructive Surgery, Haukeland University Hospital, Haukelandsveien 22, 5021 Bergen, Norway; 2 Department of Clinical Medicine, University of Bergen, Jonas Lies vei 87, 5021 Bergen, Norway; 3 Department of Plastic and Reconstructive Surgery, Sahlgrenska University Hospital, Gröna Stråket 8, SE-413 16 Gothenburg, Sweden; 4 Department of Clinical Sciences, Faculty of Medicine, Lund University, Box 117, SE-221 84 Lund, Sweden; 5 Department of Clinical Sciences, Sahlgrenska Academy, Gothenburg University Hospital, Gröna stråket 8, SE-413 16 Gothenburg, Sweden

**Keywords:** Suturing skills, Assessment tool, Technical skills assessment, Surgical education, Undergraduate training, Microsurgery, Plastic surgery

## Abstract

**Background:**

In recent years, emphasis has been put on that medical student should demonstrate pre-practice/pre-registration core procedural skills to ensure patient safety. Nonetheless, the formal teaching and training of basic suturing skills to medical students have received relatively little attention and there is no standard for what should be tested and how. The aim of this study was to develop and validate, using scientific methods, a tool for assessment of medical students’ suturing skills, measuring both micro- and macrosurgical qualities.

**Methods:**

A tool was constructed and content, construct, concurrent validity, and inter-rater, inter-item, inter-test reliability were tested. Three groups were included: students with no training in suturing skills, students who have had training, plastic surgery.

**Results:**

The results show promising reliability and validity when assessing novice medical students’ suturing skills.

**Conclusions:**

Further studies are needed on implementation of the instrument. Moreover, how the instrument can be used to give formative feedback, evaluate if a required standard is met and for curriculum development needs further investigation.

Level of Evidence: Not ratable.

## Introduction

Traditionally, surgical skills have been taught through apprenticeship in the operating room, that is, through observing an experienced doctor performing a procedure and then performing the procedure on patients under supervision. However, during the last two decades, simulation training has gained ground [1]. Simulation consists of teaching and training in a structured setting that reproduces features of the clinical setting [1]. It allows the learner to repeat and practice specific tasks and makes it possible to use an objective tool for assessment of skills and structured constructive feedback to the learners [2]. Hence, errors can be identified, analyzed, and corrected, in order to improve surgical efficacy and quality, and ameliorate ethics of surgical training, standard of care, and patient safety [1, 2]. Indeed, in recent years, a greater emphasis has been put on that medical student should demonstrate pre-practice/pre-registration core procedural skills to ensure patient safety [3]. Nonetheless, the formal teaching and training of basic suturing skills to medical students have received relatively little attention and there is no standard for what should be tested and how [3].

Assessment tools for procedural skills have to be valid and reliable [2]. Examples of existing validated assessment tools for suturing skills are the University of Western Ontario Microsurgery Skills Acquisition/Assessment instrument (UWOMSA) [4–6] and the Objective Structured Assessment of Technical Skill global rating scale OSATS [2, 7–9]. Both instruments are developed for surgeons in training. UWOMSA specifically evaluates microsurgical suturing skills and comprises three categories: quality of knot, efficiency, and handling. The learner is scored in each category on a 5-point Likert scale and a global score is calculated, with a maximum of 15 [4–6]. OSATS evaluate macroscopic suturing skills in seven domains: respect for tissue, time and motion, instrument handling, knowledge of instruments, use of assistants, and knowledge of specific procedure. The learner is scored in each category on a 5-point Likert scale and a global score is calculated, with a maximum of 35 [2, 7–9]. There are few assessment tools for suturing skills validated for medical students [2, 3]. Moreover, there are no instruments evaluating both micro- and macrosurgical qualities.

The aim of this study was to develop and validate, using scientific methods, a tool for assessment of medical students’ suturing skills, measuring both micro- and macrosurgical qualities.

## Material and methods

### Subjects and controls

Subjects and controls were recruited in September 2015. Inclusion criteria were student in the pre-clinical part of his/her medical studies, no former experience with suturing, neither in vivo nor in vitro. Exclusion criteria were inability to give informed consent to participate in the study and inability to attend the course. An e-mail with information on the study was sent to all students in the pre-clinical years of the undergraduate medical programme at the University of Bergen. The first 15 students who answered the email and met the inclusion criteria were included in the study as subjects. The number of subjects was 15 based on the use of similar amount in resembling trials [2]. By random selection, five of the 15 students were also included in a pre-course test group. In addition, a group of five practicing specialists in plastic surgery were recruited among our colleges in the department as controls, called expert controls.

### Teaching and suturing training

The teaching was set up as the standard suture skills course, normally given as part of the third year undergraduate medical training at our university. First, the subjects received a 45-min theoretical lecture covering the basic knowledge of suturing, stressing aspects such as tissue treatment, knot-tying technique, and instrument handling. A plastic surgeon then demonstrated correct suture technique and the tasks. Subjects were given 90 min to practice under supervision.

### Tasks

The tasks were to perform a simple cutaneous interrupted suture with a square knot and then a continuous cutaneous three stitch long over-and-over suture. All subjects and controls used the same types of needle holders, forceps, scissors, 3.0 Nylon sutures, and a foam suture pad.

### Development of the in-house assessment tool

An assessment tool was developed in-house, including both previously used macrosurgical [10] and microsurgical [4] quality indicators. Eight yes or no questions were included, that is, if the subject: grabs the needle with the instruments (and not with the fingers), ties a correct squared knot, holds the forceps correctly, grabs the suture with the instruments in a correct fashion (in a way that does not potentially lead to suture breakage), penetrates the foam suture pad with a 90 degrees angle, manages the suture without tangling the ends in the knot, damages the foam suture pad, and makes a parallel suture (equal length from the wound edge and equal depth on both sides). In addition, amount of time needed to complete the two different tasks was measured in seconds. As an independent control question, “a veto question” [11], the evaluators were asked to make an overall dichotomous judgment if the subject is globally able to suture or not.

### Filming process

Subjects were filmed individually performing the suturing tasks in a separate room, with no interference or guidance from the people filming. All tasks were filmed with two cameras in the same position. The field of view was the simulated operating table and the subjects gloved hands. All of the subjects were filmed after completing the teaching and training. In addition, five of the subjects were first filmed before the teaching. The control groups of plastic surgeons were filmed after receiving a description of the tasks to be performed.

### Evaluation of the films

Three independent experienced specialists in plastic surgery rated the video recordings. They were sent 25 video clips with muted sound, each showing a subject or a control performing the task. The assessors were blinded to the subjects’ identity and also wether the task was performed by a novice user or by a plastic surgeon or before or after the course. The plastic surgeons rated the participants according to the developed instrument, the knot-tying module of the University of Western Ontario Microsurgery Skills Acquisition/Assessment instrument (UWOMSA) [4–6] and the Objective Structured Assessment of Technical Skill global rating scale (OSATS) [2, 7–9]. A month after the first assessment, the assosors were sent the five films again, randomly chosen, and then made a new assessment with the same tools.

### Development of total score for the assessment tool

To calculate a total score for the developed instrument, a previously published formula was used [12, 13]: cutoff time (seconds) − completion time (seconds) − (10 × sum of errors). As nine different variables were evaluated, a maximum of nine errors could be committed. The calculated time was the total time used for both exercises (completion time simple suture + completion time continuous suture).

### Validation process and statistics

Data distribution was reviewed graphically by residual QQ plots. When the assumption of normality could be accepted, parametric tests were used and data values are given as means and standard deviations. Non-normal data were analyzed by non-parametric tests and values are given as frequencies, medians and ranges. Details of specific analysis are given in Table 1. Cutoff times for the two tasks were calculated so that 67% of the post-course subjects fell within it. Average scores and number of errors by the three assessors were calculated and used to make comparisons between different groups.

**Table 1:** Variables investigated and statistical tests used

Concept and definition	Methodology	Statistical test
Content validity		
Extent to which a test measures the intended content [2]	Review of literature on previous assessment tools	
Construct validity		
Extent to which a test is able to differentiate between a good and a bad performer [2]	Difference of scores between (1) subjects pre- and post-course and between (2) post-course subjects and expert controls	1) Paired *t* test 2) Two-sample *t* test with unequal variances
Concurrent validity		
Extent to which the results of a test correlate with gold standard tests known to measure the same domain [2]	Correlation of subjects’ in-house tool scores with their OSATS and UWOMSA scores	Spearman R non-parametric correlation
Inter-rater reliability		
Extent of agreement between more than two assessors [2]	Correlation of score given by the three different assessors	Intra class correlation (ICC) coefficients estimation of variances
Inter-item reliability		
Extent to which different components of a test correlate [2]	Correlation of in-house scores and global “able to suture” assessment	Logistic regression with AUC. The regression estimates the likelihood of “ability to suture”-judgment by an assessor and the AUC estimates the likelihood of a subject categorized as “able to suture” having the highest score, when randomly compared to a subject “not able to suture”
Inter-test reliability		
Ability of a test to generate similar results when applied at two different time points [2]	Comparison of score as assessed by the same assessor at two different time points	Repeatability coefficient (CR), intra class coefficients (ICC). The CR is computed on the basis of mean variance of all subjects as scored by all three assessors. The CR suggests that 95% of repeated scores of the same subject can be expected to differ by less than the calculated value (Vaz, Falkmer et al. 2013).

## Results

### Video assessment and total score for the in-house assessment tool

**Fig. 1:**
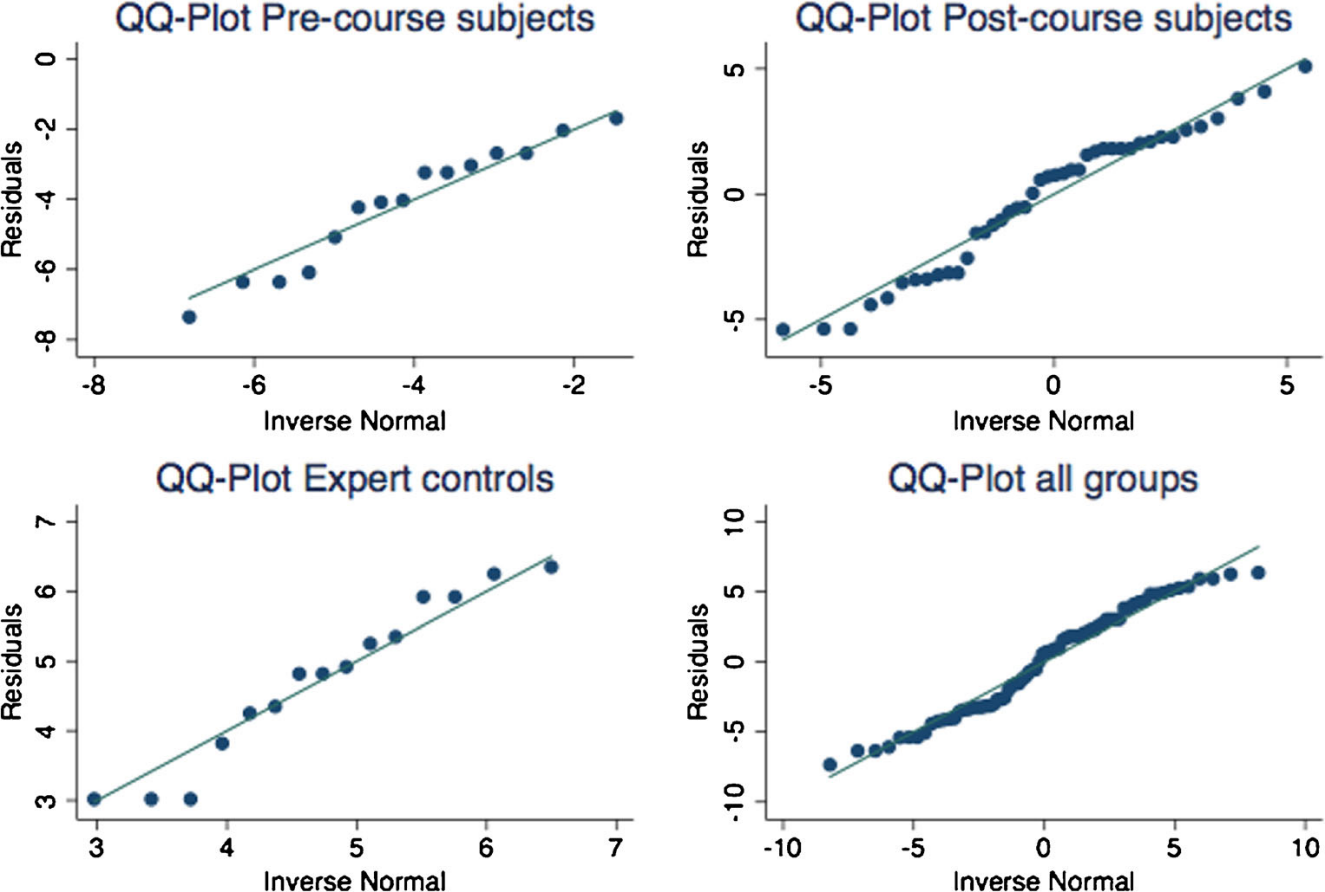
Data distribution of in-house scores by all assessors in the different groups

**Fig. 2:**
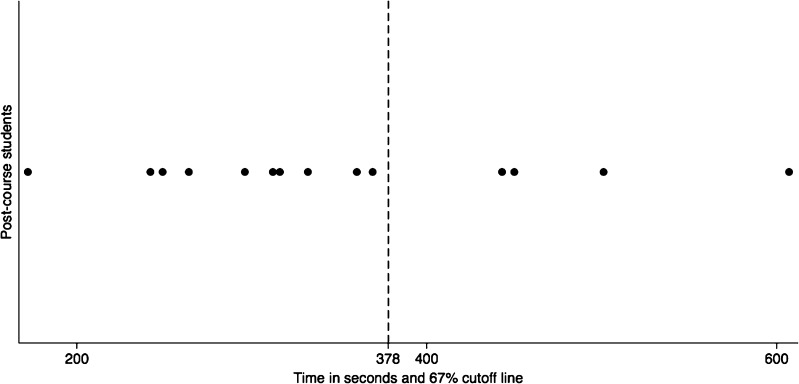
Subjects time distribution in time to complete task. The 67% cutoff is marked as the dashed line at 378 s

One of the 15 subjects who was filmed only post-course was excluded due to poor video quality. Hence, there were 14 subjects (5 pre-course and 14 post-course) and 5 expert controls. Among the remaining 14 subjects, there were 10 women and 4 men, with an age span of 19–26 years. The assessors’ video evaluation revealed that the variable “deformed needle after test” was difficult to evaluate and therefore, it was excluded from the tool and the total numbers of possible errors became eight. Data distribution of in-house scores allowed for assumption of normality (Fig. 1). Cutoff times, calculated so that 67% of the post-course subjects fell within in it, were 88 s for the simple suture and 290 s for the continuous suture, thus 378 s to complete the task (Fig. 2). The expert controls performed the task quicker than the post-course subjects (*p* < 0.01). In spite of a lower median time in post-course subjects than pre-course subjects, no real difference in time to perform the tasks was seen (*p* = 0.55) (Table 2). One of the assessors evaluated that 2/14 could not suture, one 7/14, and one 9/14.

**Table 2:** Summary statistics on performance by study group

Study group	Time to complete task (in seconds)				Number of errors registered*				In-house score*			
	Range	Median	Mean	SD	Range	Median	Mean	SD	Range	Median	Mean	SD
Pre-course subjects	279–559	372	387	112	5–6	6	6	0.3	− 1.2–3.2	2.0	1.4	1.7
Post-course subjects	172–607	324	351	116	0–5	2	2	1.5	0.8–9.3	6.3	5.3	2.7
Expert controls	97–177	123	126	32	0–2	1	1	0.6	8.6–11.1	10.8	10.3	1.1

*SD* standard deviation

*For simplicity presented as product of average score from all three assessors

### Construct validity

**Fig. 3:**
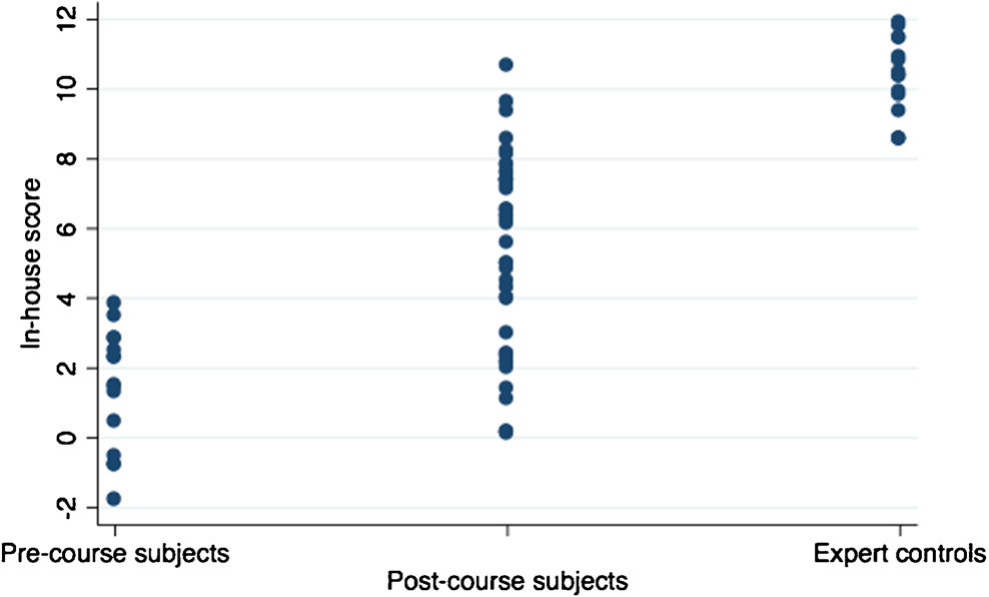
Differences in in-house scores between different groups. Individual scores from all three assessors are plotted

**Fig. 4:**
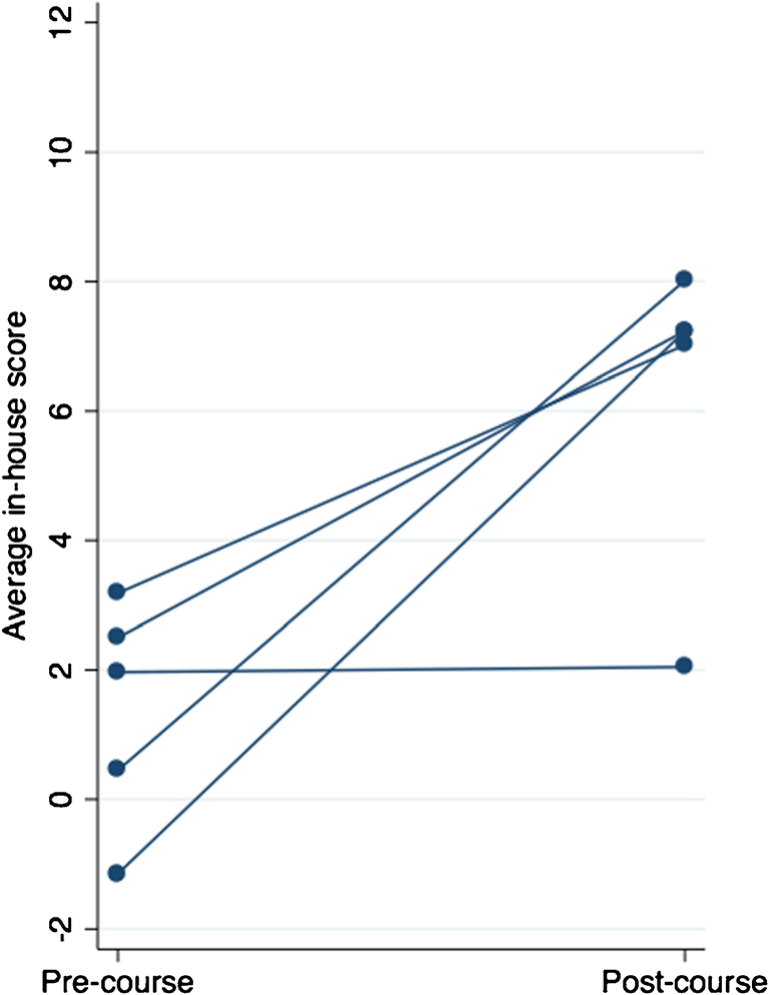
Matched improvement of pre- and post-course performance as measured by the in-house scoring tool

The in-house assessment tool was able to detect a difference between a good and a bad performer, that is between subjects pre- and post-course and between post-course subjects and expert controls (Fig. 3, Table 3). The tool’s ability to measure improvement from pre- to post-course performance in the five students who did the pre-course test is shown in Fig. 4.

**Table 3:** Summary of statistical tests of validity and reliability

Statistical test and subgroups of analysis	Output	*P*	SD	95% CI
Construct validity	md			
Paired *t* test (pre- vs post-course performance)	4.9	0.03	3.3	0.8–9.0
Two-sample *t* test (students vs experts)	5.0	< 0.01	4.0	3.9–6.0
Concurrent validity				
Spearman R non-parametric correlation				
OSATS correlation to in-house score	*ρ*			
Assessor 1	0.89	< 0.01		
Assessor 2	0.88	< 0.01		
Assessor 3	0.86	< 0.01		
Combined average	0.90	< 0.01		
UWOMSA correlation to in-house score				
Assessor 1	0.91	< 0.01		
Assessor 2	0.87	< 0.01		
Assessor 3	0.86	< 0.01		
Combined average	0.91	< 0.01		
Inter-rater reliability				
Intra class correlation (ICC) coefficients	ICC			
Pre course subject	0.83	< 0.01		0.43–0.98
Post-course subject	0.80	< 0.01		0.60–0.92
Expert controls	0.65	< 0.01		0.15–0.95
All groups combined	0.92	< 0.01		0.84–0.96
Inter-item reliability	OR (AUC)			
Logistic regression with AUC				
Assessor 1	2.68 (0.94)	0.01		1.23–5.84
Assessor 2	1.71 (0.91)	0.01		1.15–2.55
Assessor 3	2.96 (0.97)	0.04		1.07–8.22
Inter-test reliability	CR (SEM)			
Repeatability coefficient (CR)	2.7 (0.98)			
	ICC			
Intra class correlation (ICC) coefficients	0.93	< 0.01		0.79–0.99

*SD* standard deviation, *CI* confidence interval, *OR* odds ratio, *md* mean difference, *AUC* area under the curve, *ρ* Spearman’s correlation coefficient, *P p* value, *SEM* standard error of measurement (intra observer standard deviation)

### Concurrent validity

**Fig. 5:**
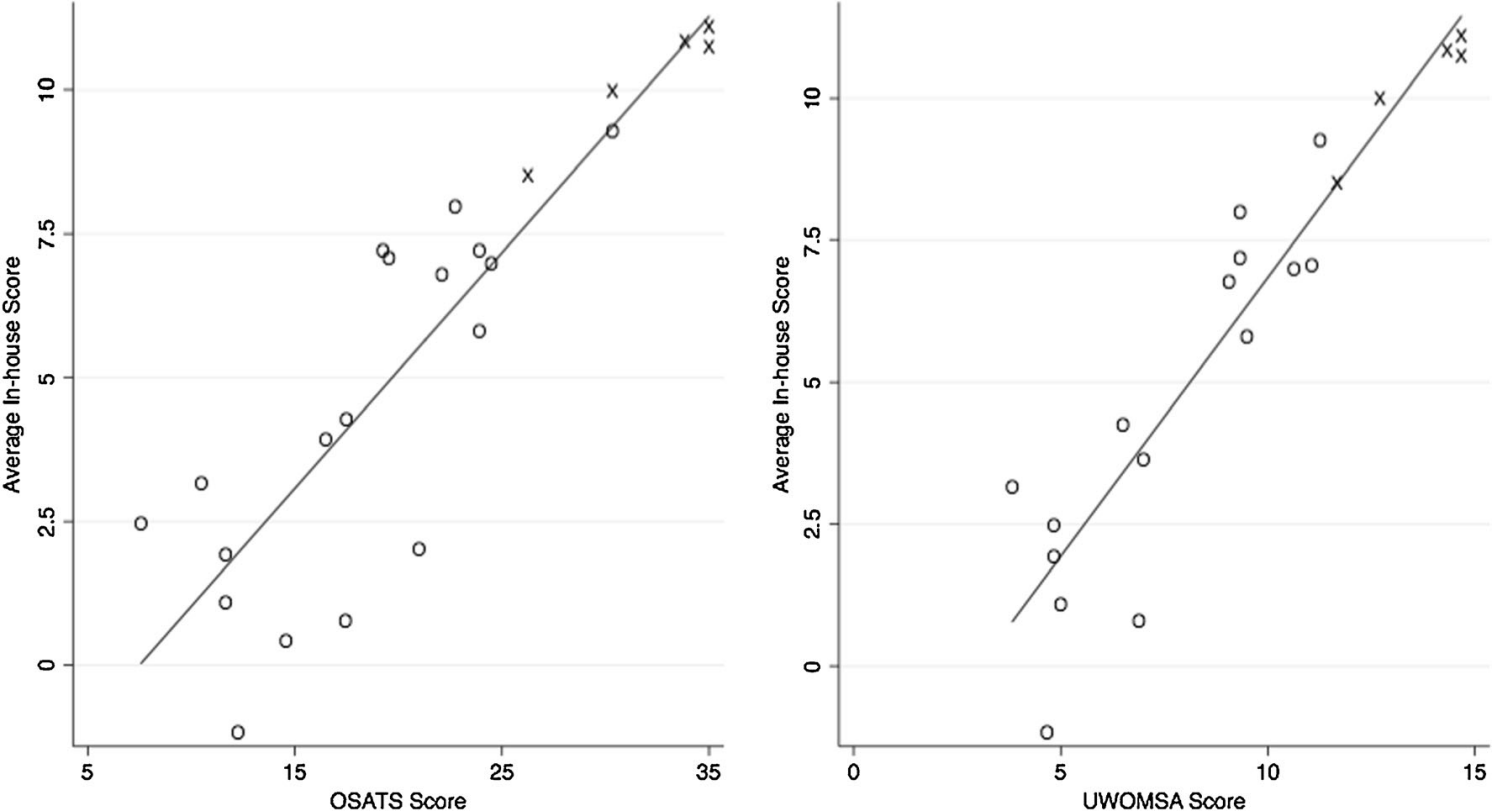
Correlation of subjects’ in-house scores with their OSATS and UWOMSA scores. O—pre- and post-course subjects, X—expert controls

An acceptable correlation was seen, for all three assessors, between the in-house total score and OSATS and OWOMSA (Table 3, Fig. 5).

### Inter-rater reliability

**Fig. 6:**
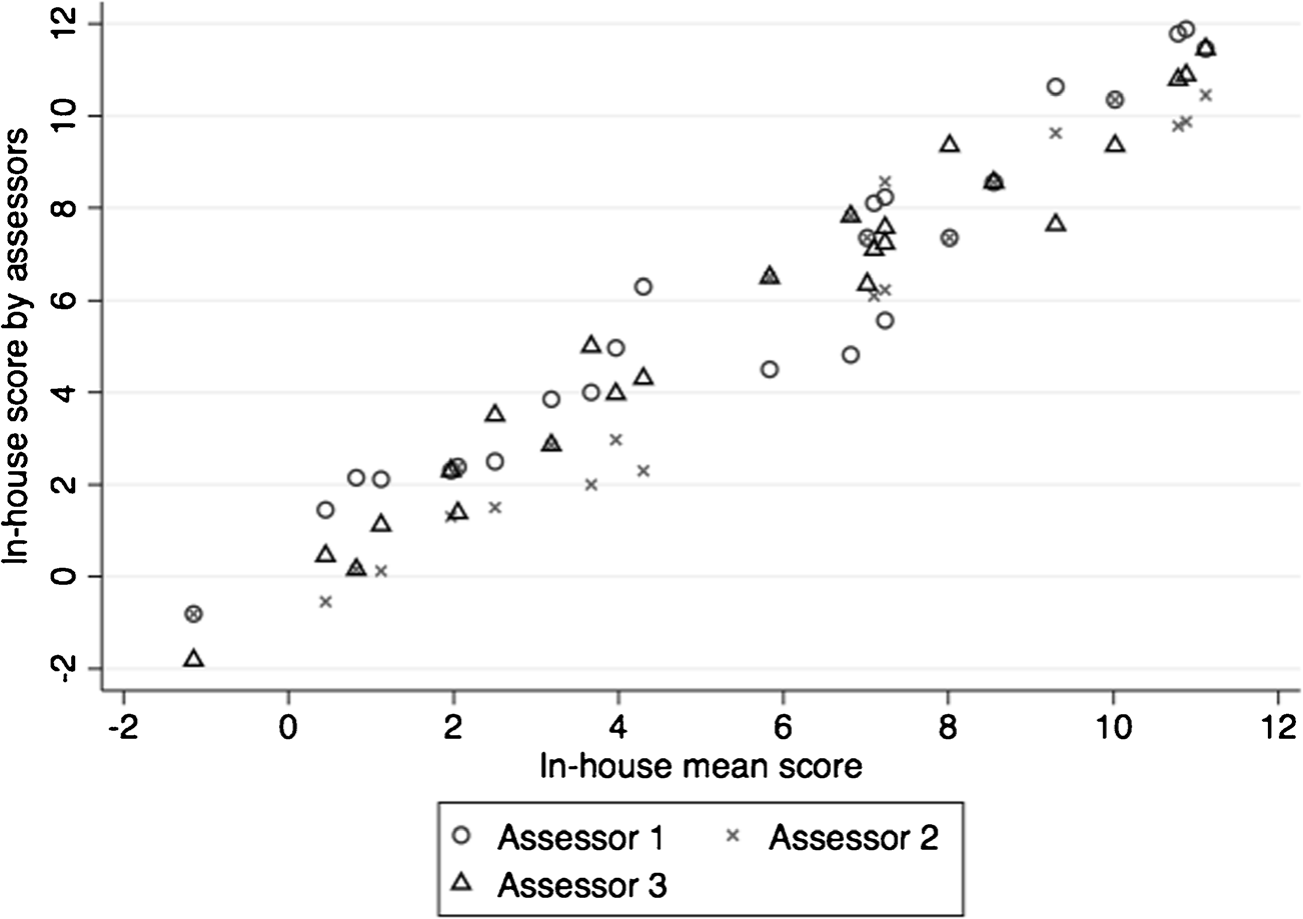
Relationship of in-house scores given by the three assessors plotted against the average score

There was an acceptable inter-rater reliability between the assessors’ in-house scores for all the study groups combined as well as for the different subgroups (Table 3). The variance accounted for by subjects was 6.63 (SD 2.58), by assessors 0.00, and residual variance of the test was 1.62 (SD 1.27). When plotting the three assessors scoring of each subject against, the mean of these three scores, no single assessor seems to score systematically different from the others (Fig. 6).

### Inter-item reliability

The likelihood for the overall assessment “able to suture” was increased with assessors’ increasing in-house score. Area under the curve (AUC) calculations revealed that the in-house scores provided a good discrimination of these ability predictions (Table 3). The inter-item reliability therefore has to be considered good.

### Inter-test reliability

**Fig. 7:**
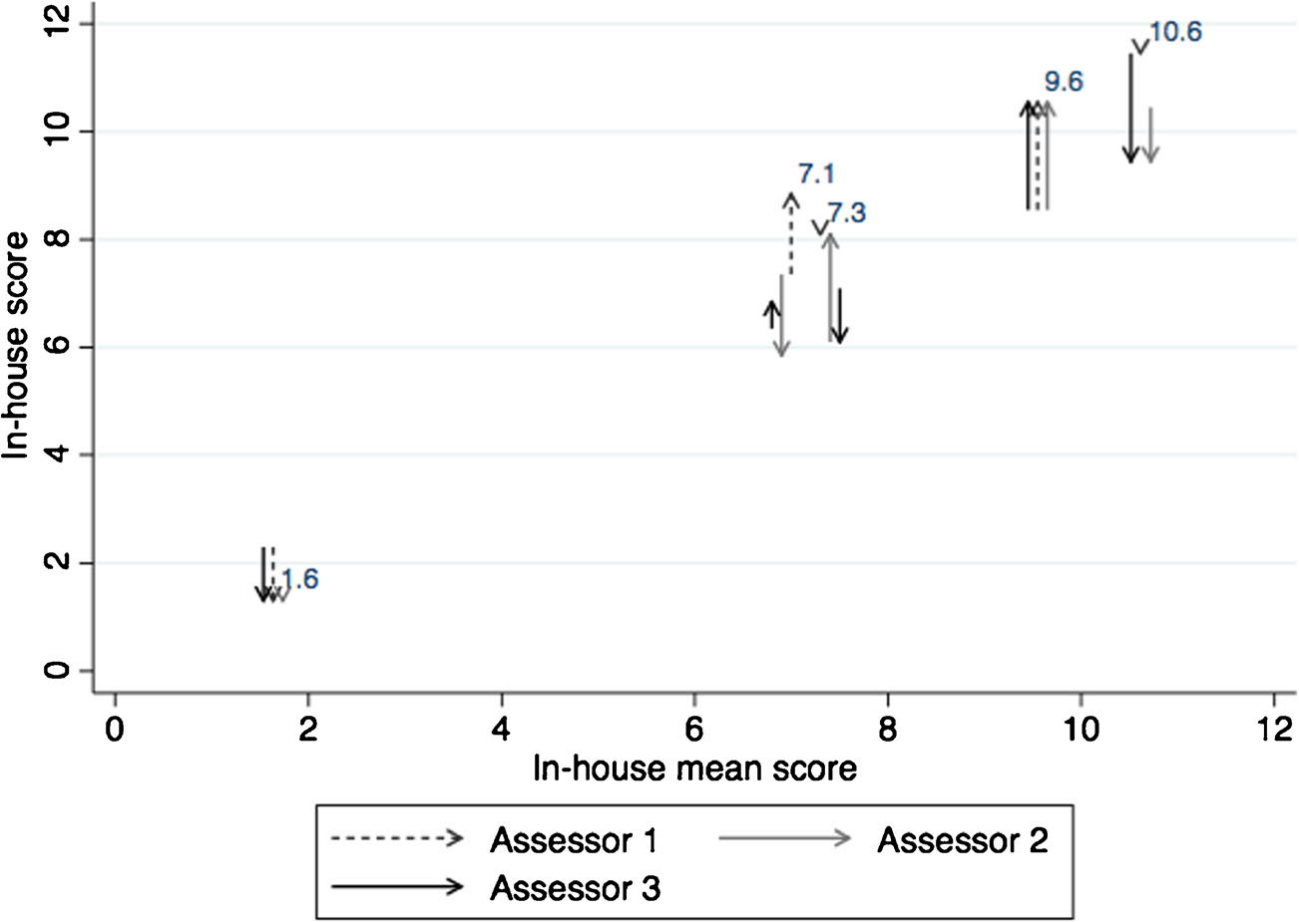
Repeatability of the in-house score. Single assessor scores plotted against the average score by the three assessors. Arrows represent scores of the same subject by the same assessor at two different time points. The starting point of the arrow is the score given at the first assessment and the arrowhead represents the second assessment score. Large differences in the first and second scores by the same assessor can be spotted as elongated arrows. Only arrowheads are shown when the two scores are equal. The average score is indicated as a numeric value on the graph

None of the assessors had systematically differences in scores between assessment 1 and 2 (Fig. 7). The calculated repeatability coefficient (CR) suggests that 95% of repeated scores of the same subject can be expected to differ by less than 2.7 points (Table 3). This quantifies the repeatability of the in-house tool. The ICC showed a good reliability of repeated scores (Table 3).

## Discussion

There are few studies on assessment tools for medical students’ suturing skills [2, 3]. This is a study that develops and validates an assessment tool for suturing skills, measuring both micro- and macrosurgical quality indicators, in medical students.

There is no consensus on how reliability and validity should be measured [14] and the statistical methods chosen can affect the result. For instance, correlation coefficients are typically used to describe reliability, but their weakness is the lack of quantifying agreement and their insensitivity to systematic measurement errors [15, 16]. Because correlation only measures how closely a set of paired observations follow a straight line and not the agreement, a correlation analysis could show a close to perfect correlation, but still be diverging from true values, and thus be misleading. On the other hand, intra class correlation (ICC), comparing more than two sets of measurement, has the strength of accounting for within subject variability and average variability, but is highly influenced by the homogeneity of data [15]. This could explain that the lowest ICC score of inter-rater reliability are in the expert control group (Table 3), even though this group has the closest range of scores of all three study groups (Table 2 and Fig. 2). Methodological researchers have advocated the use of repeated measurements to compare agreement between methods and the agreement of a method to itself and thereby quantifying disagreement [16]. This way, agreement is not only present or absent, but quantified. In the present study, there were no previous golden standard tools with identical scale of measurement that the new tool could be validated against. Accuracy measurements by repeatability were therefore only conducted for comparison of the assessors’ in-house scores of the same subjects at two different time points (inter-test reliability), as this was the only available option for repeated measures (Table 3). However, the assessors were blinded to the fact that they were producing repeated measurement of the exact same tests twice. This was possible due to the fact that tests were assessed as films, contrary to live assessment. Thus, the calculated CR might represent the most appropriate measurement of the accuracy of the test, and whether the test has sufficient accuracy for future purposes can be extrapolated.

The results might be affected by the composition of the sample. In this study, only students who had no previous experience with suturing, neither in vivo nor in vitro, were included and hence, they were all truly novices. Therefore, differences in previous experiences cannot be considered a factor. It can be questioned whether our sample is representative of medical students or may be comprised of students who are extra interested in acquiring suturing skills or are extra apt for surgery. However, the students were their own controls, or tested against experienced plastic surgeons, when the construct validity was tested and hence the subjects’ aptitude for or interest in surgery should not have affected the results.

Evaluation of films of the task performed by the subjects, using checklists, has been done in earlier studies similar to this one [4, 17]. Several benefits have been found with evaluations of films rather than a live performance [4]. For instance, it is possible to blind the assessor the identity of the subject and, in this case, if the performance is pre- or post-course, it makes it possible for several assessors to evaluate the performance simultaneously [4], and for the assessors to rewind or fast forward as they need [11]. A possible confounder is that the evaluations could be affected by assessor’s fatigue when a large number of tapes have to be watched and analyzed. In order to minimize this risk, the sample size was kept small and the assessors were not constrained to a certain deadline. The small variations seen between assessors are inherent as there always is a touch of subjectivity in any evaluation of a performance. Even though a checklist is used, assessors might find some quality indicators more or less important than others, and therefore be more or less harsh in their evaluation. For example, this might explain why one of the assessors seemed to be more accepting than the other two when making the overall evaluation of if the subject could suture or not.

Skill proficiency is difficult to define for suturing, especially at undergraduate level and an assessment tool needs to be able to capture different aspects, as well as giving an overall evaluation. Time alone is a bad measurement as it does not take quality into consideration [18] and as novices might sometimes not be aware of all steps of a procedure, they might take shortcuts leading to fast procedure times, but poor results [17]. Furthermore, the instrument was able to detect a difference between pre- and post-course performance (*p* = 0.03) (Fig. 4), whereas there was no detectable difference in time used (*p* = 0.55), indicating that time is not sensitive enough. On the other hand, time has to be part of the assessment, as proficiency not only is characterized by a good end result but also of efficiency. In previous studies, time has also been incorporated in the overall assessment in different ways, either as a measurement of time taken to complete a task [12] or as number of tasks performed during a certain time [19]. We calculated the cutoff times for the tasks so that 67% of the post-course subjects fell within in it (Fig. 2). In previous study, the authors have not stated how the cutoff time was assigned [12]. As most of the subjects falling within the 33% have to be considered outliers (Fig. 2), we consider this an adequate cutoff time. The number of errors was weighted by a factor of 10, as previously described [13], to emphasize the importance of a correct suture technique and good quality knot, in relation to time used. Nonetheless, how time and errors are weighted is arbitrary, but the model used in this study has been successfully utilized in other studies [12, 13]. The strength of this new instrument is that it evaluates quality indicators important to both micro- and macrosurgical quality indicators and in addition to the total score, individual qualities can be analyzed specifically.

An assessment tool for suturing skills in medical students can be useful both to give formative feedback to the students [20, 21] as well as to evaluate if the students meet the required standard [11], and for curriculum development [19, 22–25]. Further studies are needed to evaluate how our instruments can be used for these purposes. Moreover, studies are needed on the implementation of the instrument, that is, on the feasibility, acceptability, educational impact and effectiveness of the instrument [2] and on the transferability to the clinical environment (face validity) [1, 2].

In conclusion, our findings suggest that the developed in-house assessment tool shows promising reliability and validity when assessing novice medical students’ macroscopical suturing skills. Further validation is needed for microsurgical suturing skills.
